# Mediterranean Diet and Global Dietary Shifts, 1990–2018: Population-Level Analysis Using Global Dietary Database

**DOI:** 10.3390/nu18182943

**Published:** 2026-09-08

**Authors:** Dana Popescu-Spineni, Leila Karimi, Laima Brazionis, Elena Philippou, Audrey Tierney, Jelena Helene Cvejić, Linda Errington, Labros Sidossis, Catherine Itsiopoulos

**Affiliations:** 1 Department of Social Medicine, Faculty of Midwives and Nursing, “Carol Davila” University of Medicine and Pharmacy, “Francisc I. Rainer” Institute of Anthropology, Romanian Academy, 050711 Bucharest, Romania; spineni.popescu@umfcd.ro; 2 Mediterranean Lifestyle Medicine Institute, 85400 Leros, Greece; leila.karimi@rmit.edu.au (L.K.); audrey.tierney@ul.ie (A.T.); jelena.cvejic@mf.uns.ac.rs (J.H.C.);; 3 School of Health and Biomedical Sciences, STEM College, RMIT University, Melbourne 3000, Australia; 4 School of Medicine and Healthcare Management, Caucasus University, Tbilisi 0102, Georgia; 5 Diabetes and Vascular Medicine Laboratory, Baker Heart and Diabetes Institute, Australia and Baker Department of Cardiometabolic Health, University of Melbourne, Melbourne 3004, Australia; 6 Department of Life Sciences, School of Life and Health Sciences, University of Nicosia, 2417 Nicosia, Cyprus; 7 Department of Nutritional Sciences, King’s College London, London SE1 9NH, UK; 8 School of Allied Health and Centre for Implementation Research, Health Research Institute, University of Limerick, V94 T9PX Limerick, Ireland; 9 Biomedical Sciences Group, Translational Research Center in Gastrointestinal Disorders (TARGID), Department of Chronic Diseases and Metabolism, KU Leuven, 3000 Leuven, Belgium; 10 Department of Pharmacy, Faculty of Medicine, University of Novi Sad, 21000 Novi Sad, Serbia; 11 Walton Library, School of Biomedical, Nutritional and Sport Sciences, Newcastle University, Newcastle upon Tyne NE2 4HH, UK; 12 Department of Kinesiology and Health, School of Arts and Sciences, Rutgers, The State University of New Jersey, New Brunswick, NJ 08901, USA

**Keywords:** Mediterranean diet, nutrition transition, global dietary shifts, Global Dietary Database

## Abstract

**Background**: The Mediterranean diet (MD) has well-established health benefits, yet modern food systems are driving a nutrition transition. Quantifying how Mediterranean countries have shifted relative to global trends is essential for evidence-based public health. **Aim**: Dietary changes between 1990 and 2018 were examined in Mediterranean coastline countries (MED) in comparison with high-income non-Mediterranean countries (HIC non-MED) and middle-/low-income non-Mediterranean countries (MLIC non-MED). **Methods**: Using the Global Dietary Database, within-country changes (1990 vs. 2018) for 13 food categories were analysed across 185 nations in three groups: MED countries (*n* = 21), HIC non-MED (*n* = 18), and MLIC non-MED (*n* = 146). MED countries were further grouped into Southern Europe (SE), Western Asia (WA), and North Africa (NA). Paired unweighted country-level analyses were the primary analyses; population-weighted analyses were sensitivity checks. Benjamini–Hochberg’s false-discovery-rate correction and centred log-ratio compositional analysis were applied. **Results**: MED seafood intake rose from 20.5 to 33.8 g/day (Δ = +13.3 g/day; 95% CI: +6.2 to +18.4; q = 0.003), and non-starchy vegetables increased from 135.5 to 173.6 g/day (Δ = +38.0 g/day; q = 0.626). Refined/starchy carbohydrate intake rose by 63.3 g/day in MED countries while declining by 33.6 g/day in HIC non-MED countries (between-group divergence +96.9 g/day; q = 0.006). Nuts and seeds rose across all groups; legumes declined modestly in MED countries. Saturated fatty acids + omega-6 polyunsaturated fatty acids (% kcal) rose by +0.6 percentage points in MED countries but fell in HIC non-MED countries. Monounsaturated fatty acids were stable. MED-WA showed the greatest overall divergence from the SE 1990 pattern. **Conclusions**: These findings highlight a public health paradox: the MD is globally promoted as a model of healthy eating, yet adherence within its region of origin is declining. Targeted, region-specific strategies are urgently needed to preserve beneficial MD elements while addressing emerging diet-related risks.

## 1. Introduction

The Mediterranean diet (MD), rooted in a region with over 5000 years of recorded history, was recognised by UNESCO as an intangible cultural heritage of humanity [1,2] and is widely acknowledged for its long-term health benefits and sustainability [3,4]. Given that this region is home to some of the world’s oldest cultures, with strong bonds formed through centuries of commerce and communication across a common sea and an often-challenging natural environment, the MD is also known for its “dietary polymorphism” [5,6]. The Mediterranean diet is characterised by high consumption of vegetables, fruits, legumes, whole grains, nuts, and olive oil as the primary fat source; moderate intake of fish, seafood, and dairy; and low consumption of red and processed meat and added sugars [7]. Although the term “Mediterranean diet” suggests a common dietary pattern, cultural, economic, and geographical factors result in important differences across Mediterranean countries and within them [4,8].

Being deeply intertwined with Mediterranean lifestyles, the Mediterranean diet has been substantially shaped by cultural and economic changes over recent decades. In particular, the Mediterranean region, like many parts of the world, is experiencing a nutrition transition towards more westernised dietary patterns, characterised by greater reliance on industrially produced and ultra-processed foods [9,10].

Paradoxically, even as the Mediterranean diet gains global popularity and increasing recognition from the scientific community, Mediterranean populations are moving further away from this traditional dietary model [9,11,12,13,14]. Changes in diets and consumption patterns in the Mediterranean region reflect interconnected factors, including population growth, urbanisation, globalisation, the homogenisation of lifestyles, food-price volatility, and economic pressures mostly affecting poorer populations and food-importing countries.

Specifically, recent data indicate a decline in adherence to the traditional Mediterranean diet across Northern, Southern, and Eastern Mediterranean countries [11,12,13,14,15]. Major concerns include increased consumption of saturated-fat-rich foods, industrial foods, and sugar-sweetened products; reduced consumption of traditional staples such as legumes and minimally processed cereals; and a shift in protein sources, with animal proteins increasing at the expense of plant proteins [11,12,13,14,15].

These dietary shifts have important public health implications. Overweight and obesity rates continue to rise across Mediterranean countries, while some Southern and Eastern Mediterranean populations also experience the double burden of undernutrition and chronic nutrition-related diseases, such as type 2 diabetes mellitus [15,16].

Understanding these trends is crucial for promoting the traditional Mediterranean diet and supporting its longevity in diverse cultural contexts. The transferability of the archetypal Mediterranean diet’s health benefits has been demonstrated beyond its traditional borders, indicating that regional adaptations both within and beyond the Mediterranean basin can embrace its core principles [17,18].

So, despite growing recognition of the Mediterranean diet’s health benefits, there is a lack of harmonised, cross-national, long-term evidence on how dietary patterns within Mediterranean countries have shifted relative to global trends. The present study addresses this gap using the Global Dietary Database, the most comprehensive harmonised global dietary dataset currently available. In this context, this study analysed food consumption trends between 1990 and 2018 in countries along the Mediterranean coastline (MED) and compared them with high-income non-Mediterranean countries (HIC non-MED) and middle-/low-income non-Mediterranean countries (MLIC non-MED). The main research questions were as follows:

Q1—How does the within-country change (1990 → 2018) in intake of 13 selected food groups differ between MED countries, HIC non-MED countries, and MLIC non-MED countries?

Q2—Which Mediterranean sub-regions (Southern Europe (SE), Western Asia (WA), North Africa (NA)) have mostly preserved, and which have mostly moved away from, the traditional Mediterranean dietary pattern between 1990 and 2018?

## 2. Methods

Data were extracted from the 2018 Global Dietary Database (GDD), a Tufts University and Global Nutrition and Policy Consortium initiative [19]. Country-level dietary data for 185 countries were obtained for 1990 and 2018. Countries were classified into three mutually exclusive groups using fixed 2018 World Bank income criteria (World Bank, 1 July 2018) and geographic definitions: (1) MED countries (*n* = 21), (2) HIC non-MED countries (*n* = 18), and (3) MLIC non-MED countries (*n* = 146). MED countries were defined as countries bordering the Mediterranean Sea and were further grouped geographically into three subregions: Southern Europe (SE), Western Asia (WA), and North Africa (NA).

*Statistical analysis:* Country-level mean dietary intakes for the 185 GDD countries were analysed for 1990 and 2018. Each country served as its own control in a paired design, with within-country dietary changes (Δ = 2018 − 1990) calculated for the following 13 main food categories: (i) non-starchy vegetables, (ii) fruits, (iii) whole grains, (iv) legumes, (v) nuts and seeds, (vi) seafood, (vii) eggs, (viii) dairy total (cheese + yoghurt + milk), (ix) refined/starchy carbohydrates (refined grains + potatoes + other starchy vegetables), (x) free sugars and sugar-sweetened beverages (SSBs + 100% fruit juice), (xi) processed meats, (xii) monounsaturated fatty acids (MUFA), and (xiii) saturated fatty acids (SFA) + omega-6 polyunsaturated fatty acids (PUFA). No harmonised country-year estimates for unprocessed meat were available in the GDD dataset. Refined/starchy carbohydrate intake was used as a proxy for higher-glycaemic refined/starchy carbohydrate exposure and should not be interpreted as equivalent to refined grains alone. Full GDD source columns and aggregation rules for each category are tabulated in Table 1.

Weighting strategy: Two analytical specifications were used: the unweighted country-level analysis was pre-specified as the primary analysis, and a population-weighted specification was used as a sensitivity check. In the primary (unweighted) analysis, each of the 185 countries was treated as one independent observation; group and subregion summaries are the arithmetic means of the country-level Δ values. Statistical inference used one-sample paired *t*-tests on the within-country differences (Δ = 2018 − 1990) and independent-samples *t*-tests on the between-group contrasts, with Benjamini–Hochberg’s FDR correction applied separately within each test family (between-group contrasts; within-subregion paired changes) (Table S1). All q-values reported in the Abstract, Results, figure captions, and Table S2 are from this unweighted primary analysis. In the sensitivity (population-weighted) analysis, each country contributed a weight equal to its 2018 estimated population from the UN World Population Prospects as integrated in the GDD; the same weight was applied to the 1990 and 2018 paired observations. Weighted mean Δ values were computed as $\bar{\Delta }$ = Σ_i_ w_i_·Δ_i_/Σ_i_ w_i_, with 95% CIs derived from the standard weighted-variance estimator. The purpose of the weighted analysis was to confirm that conclusions drawn from country-level means were not driven by small-population outliers and that the conclusions were held at the per-person dietary level. Weighted point estimates and 95% CIs are discussed in the Results only where they alter the qualitative conclusions of the primary analysis. Population-weighted q-values are not reported in the main text to avoid implying that the two specifications are interchangeable primary analyses.

Food categories were derived from the GDD using direct mapping and composite aggregation (Table 1). For energy-normalised analyses, all g/day measures were converted to g/day per 2000 kcal using estimated total energy intake derived from macronutrient energy contributions. Because the GDD does not report animal- or plant-source fats in g/day, fatty acid intakes (MUFA, total PUFA, omega-6 PUFA and SFA) were obtained as percentages of total energy intake (% kcal). Both dietary quantity (food-group g/day) and fatty-acid composition (% kcal) were therefore included in the analysis, given the nutritional relevance of MUFA-rich foods, such as olive oil, to the Mediterranean dietary pattern. Energy validity checks excluded country-years with implausible estimates (<1200 or >4000 kcal/day).

Results are presented as mean within-country change (Δ = 2018 − 1990) with 95% confidence intervals (CIs) and, for between-group contrasts, with Benjamini–Hochberg’s false-discovery-rate-adjusted q-values. Statistical significance was set at α = 0.05 for individual tests, with FDR-adjusted significance (q < 0.05) reported for multiple comparisons. Effect sizes (standardised mean differences) are reported alongside q-values where appropriate. The q-value reported throughout this study is the Benjamini–Hochberg false-discovery-rate (FDR)-adjusted analogue of a *p*-value [20]. Because the present analysis evaluates multiple food categories across country groups and Mediterranean subregions, uncorrected *p*-values would inflate type-I error. FDR adjustment was therefore applied separately within each test family to retain statistical power while controlling the expected proportion of false discoveries. A q-value below 0.05 was taken as the threshold for statistical significance throughout the Abstract, Results and figure captions.

Note that throughout the Abstract, Results, and figure captions, all reported significance values are Benjamini–Hochberg’s FDR-adjusted q-values (q < 0.05 considered significant). Unadjusted *p*-values for every individual test are provided in Supplementary Table S1 for transparency. Cohen’s d effect sizes are also reported in Supplementary Table S2 to quantify the practical magnitude of each within-group dietary change.

Compositional dietary changes were additionally examined using a centred log-ratio (CLR) transformation. For each country and year, the 11 g/day food groups (not including the fat groups, which were treated as %kcal) were treated as a closed composition; CLR-transformed intakes (log of each group divided by the geometric mean of all 11 groups) were then differenced (Δ_CLR = CLR_2018 − CLR_1990). This compositional approach evaluates each food group’s share within the overall diet, independent of absolute caloric intake. Zero values were checked before transformation; where structural or sampling zeros would prevent log-ratio transformation, a small positive replacement value would be required. In the present analysis, no substantive interpretive conclusion was based on zero-replacement choices. Fats (% kcal) cannot be entered into a g/day CLR composition and are therefore reported on a separate, comparable % kcal scale throughout. Paired tests on Δ_CLR were used to confirm consistency with the absolute g/day results.

To capture subregional dietary trajectories across the Mediterranean basin, we applied an exploratory Population-Standardised Dietary Deviation (PSDD) framework. All dietary variables (g/day and % kcal) were z-scaled against the 1990 cross-country distribution, placing the axes on a common dimensionless scale. Southern Europe in 1990 was designated as the reference profile because it most closely represents the conventional historical reference for the traditional Mediterranean dietary pattern in the available data.

For adverse dietary components (refined/starchy carbohydrates, free sugars and sugar-sweetened beverages, processed meats, and SFA + omega-6 PUFA), axis values were sign-flipped so that outward displacement on radar figures reflects closer alignment with the reference pattern. Subregional dietary profiles and trajectories are presented in Figures 3 and 4.

All analyses were conducted in Python 3.13.9 (pandas 2.4, statsmodels 0.15).

## 3. Results

Q1: How does the within-country change (1990 → 2018) in intake of the 13 selected food groups differ between Mediterranean coastline countries (MED), high-income non-Mediterranean countries (HIC non-MED), and middle-/low-income non-Mediterranean countries (MLIC non-MED)?

Among the 13 food categories analysed, the most pronounced between-group contrast was observed for refined/starchy carbohydrates. Intake increased in MED countries from 274.78 g/day in 1990 to 338.05 g/day in 2018 (Δ = +63.27 g/day), while declining in HIC non-MED countries from 194.64 g/day to 161.06 g/day (Δ = −33.58 g/day). This divergence of +96.9 g/day between MED and HIC non-MED represented the largest statistically significant between-group difference observed (q = 0.006).

Seafood consumption showed the most favourable relative change in MED countries, increasing from 20.49 g/day to 33.79 g/day (+13.30 g/day), compared with increases from 24.91 g/day to 27.89 g/day in HIC non-MED and from 34.41 g/day to 35.59 g/day in MLIC non-MED (q = 0.003).

Non-starchy vegetables increased in MED countries from 135.53 g/day to 173.55 g/day (+38.02 g/day), compared with increases from 97.88 g/day to 116.13 g/day in HIC non-MED and from 135.80 g/day to 151.82 g/day in MLIC non-MED. This difference did not reach statistical significance after FDR correction.

Fruits increased in MED countries from 114.84 g/day to 132.21 g/day (+17.37 g/day), compared with increases from 118.94 g/day to 140.59 g/day in HIC non-MED and from 90.82 g/day to 102.21 g/day in MLIC non-MED.

Nuts and seeds increased across all three groups: MED from 4.67 g/day to 9.60 g/day (+4.93 g/day), HIC non-MED from 2.65 g/day to 5.16 g/day (+2.51 g/day), and MLIC non-MED from 4.31 g/day to 8.19 g/day (+3.88 g/day). Between-group differences did not reach statistical significance after FDR correction.

Legumes declined modestly in MED countries from 22.50 g/day to 21.66 g/day (−0.84 g/day), while declining slightly in HIC non-MED (from 15.87 to 15.41 g/day) and increasing in MLIC non-MED (from 29.79 to 31.73 g/day). None of these changes reached statistical significance after FDR correction.

Whole grains showed a modest increase in MED countries from 30.62 g/day to 36.70 g/day (+6.08 g/day), while remaining relatively stable in HIC non-MED (47.47 to 46.03 g/day) and MLIC non-MED (39.19 to 39.69 g/day).

Dairy total increased in MED countries from 192.60 g/day to 241.60 g/day (+49.00 g/day), compared with increases from 274.80 g/day to 297.77 g/day in HIC non-MED and from 123.13 g/day to 136.43 g/day in MLIC non-MED.

Eggs increased in MED countries from 20.61 g/day to 24.75 g/day (+4.14 g/day), compared with smaller increases in HIC non-MED (16.94 to 17.82 g/day) and larger increases in MLIC non-MED (16.34 to 22.62 g/day).

Processed meats increased in MED countries from 15.37 g/day to 24.63 g/day (+9.25 g/day), compared with increases from 25.82 g/day to 32.45 g/day in HIC non-MED and from 15.69 g/day to 19.80 g/day in MLIC non-MED.

Free sugars and sugar-sweetened beverages increased substantially in MED countries from 120.00 g/day to 169.62 g/day (+49.62 g/day), and in MLIC non-MED from 153.81 g/day to 201.43 g/day (+47.62 g/day), while remaining virtually unchanged in HIC non-MED (135.44 to 136.04 g/day). The MED vs. HIC difference did not reach statistical significance after FDR correction.

MUFA (% kcal) showed minimal change across all groups: MED from 13.25% to 13.10% (−0.15%), HIC non-MED from 13.22% to 13.46% (+0.23%), and MLIC non-MED from 11.47% to 10.85% (−0.62%). None of these changes were statistically significant.

**Figure 1:**
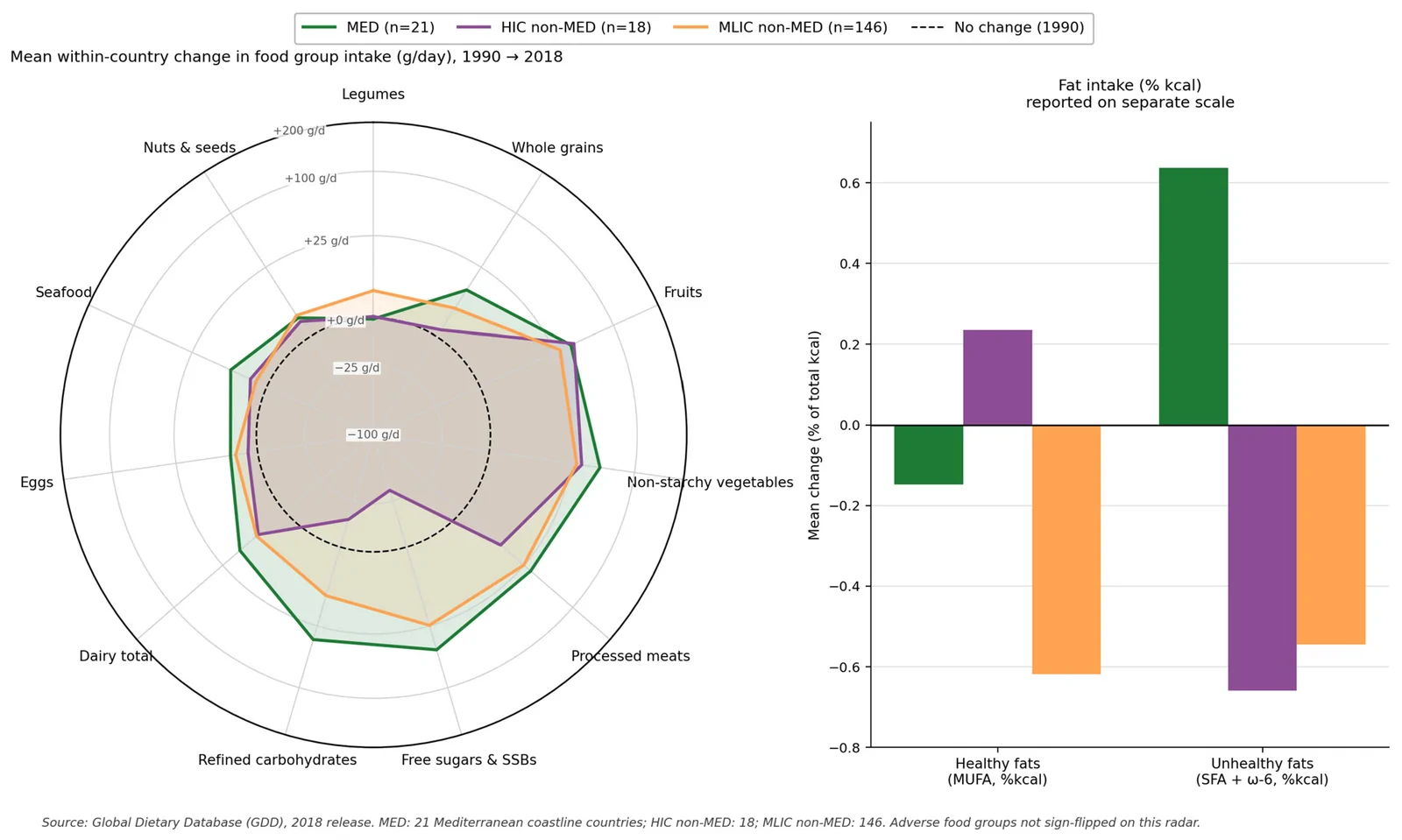
Mean change in selected groups by country group (MED, HIC non-MED, MLIC non-Med), 1990–2018. X-axis: Selected food groups (g/day; fats reported on a separate % kcal panel because units differ). Y-axis: Mean within-country change in intake (Δ = 2018 − 1990). Coloured polygons show MED (green; *n* = 21), HIC non-MED (purple; *n* = 18) and MLIC non-MED (orange; *n* = 146). Source: Global Dietary Database (GDD), 2018 release. Quantitative values, 95% Cis, and FDR-adjusted q-values for every food group are provided in the Results text above and in Supplementary Tables S1 and S2; this figure is intended as a visual summary and is not duplicated numerically in the figure legend.

SFA + omega-6 PUFA (% kcal) showed a small increase in MED countries from 13.69% to 14.33% (+0.64%), a decrease in HIC non-MED from 16.94% to 16.28% (−0.66%), and a decrease in MLIC non-MED from 13.40% to 12.85% (−0.55%). See Figure 1 for a summary.

**Figure 2:**
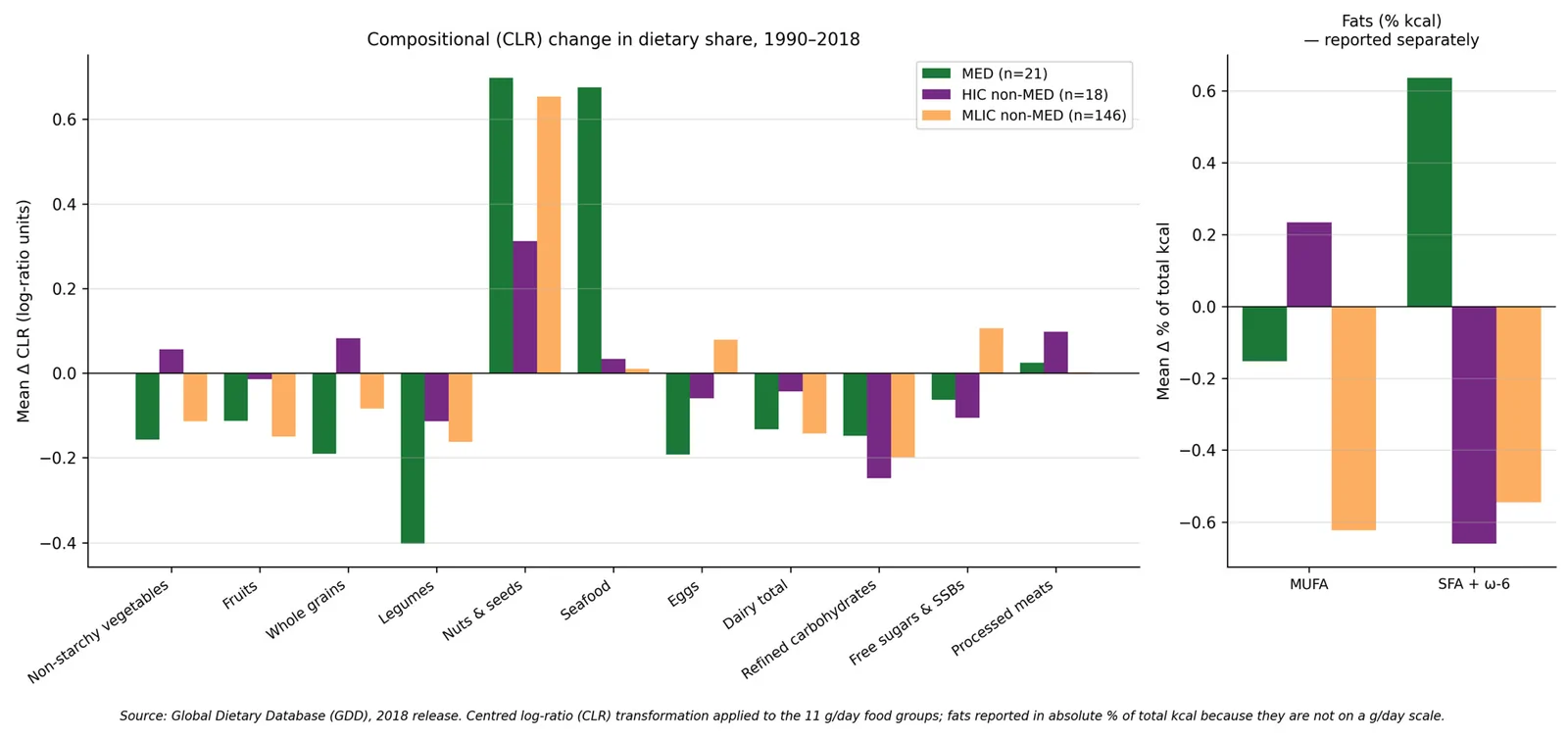
Proportional (CLR) dietary change by country group, 1990–2018.

Mean centred-log-ratio (Δ_CLR) change in the share of each of the 11 g/day food groups in MED, HIC non-MED and MLIC non-MED country groups. Refined/starchy carbohydrates, free sugars, and SSBs are shown alongside protective groups so that the full compositional shift is visible. Fats (% kcal) are presented on the right-hand panel because they are not on a g/day scale and therefore cannot be included in a closed g/day composition. Paired *t*-tests on Δ_CLR confirmed consistency with the absolute g/day results (*p*-values reported in Supplementary Table S1, see Figure 2 for a summary).

Q2: Which Mediterranean subregions (SE, WA, NA) have most preserved, and which have most moved away from, the traditional Mediterranean dietary pattern between 1990 and 2018?

**Figure 3:**
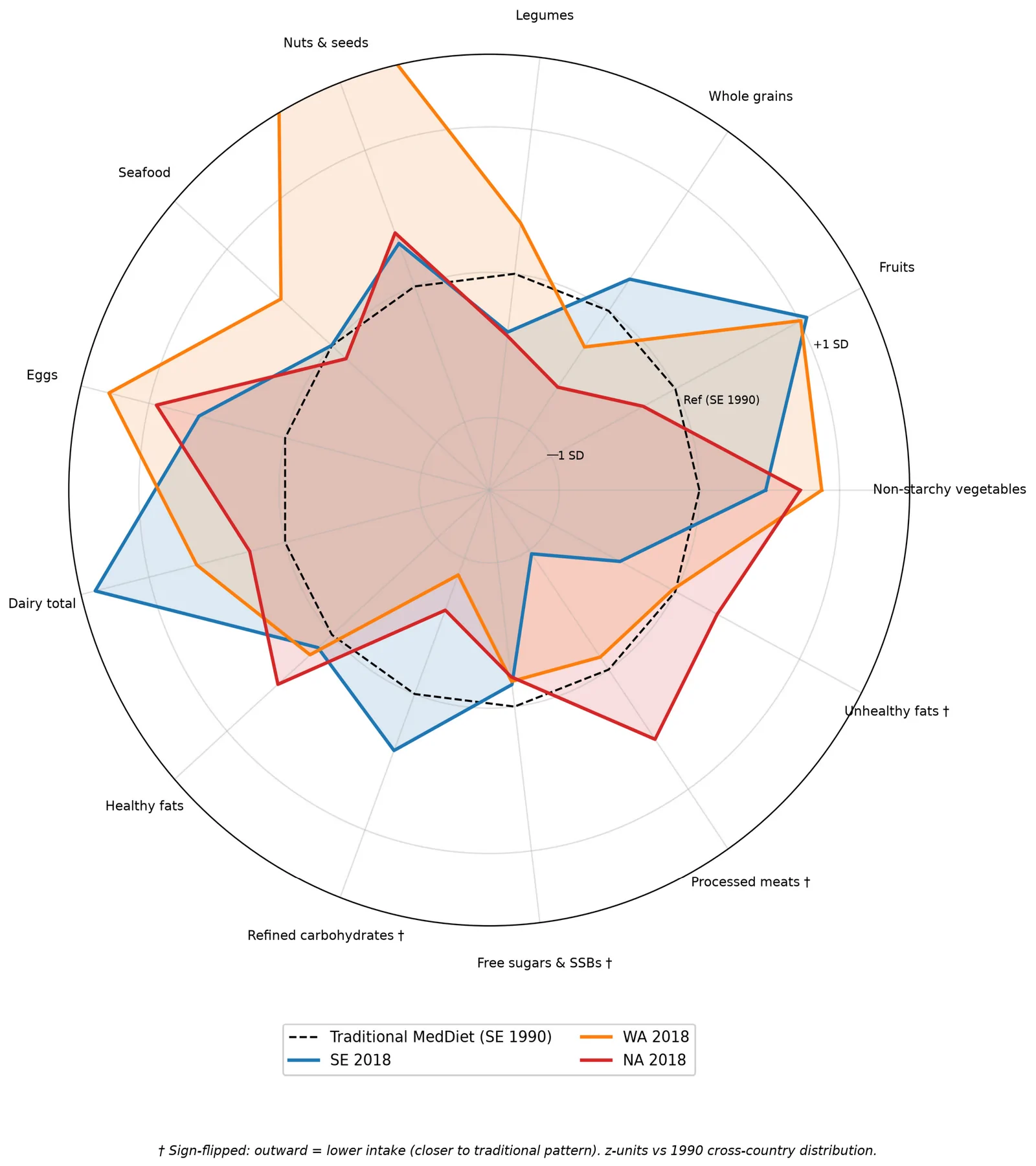
Subregional mean within-country changes in food group intakes, 1990–2018 (g/day). Southern Europe (SE) showed statistically significant increases in seafood (+13.7 g/day; 95% CI: 5.9 to 21.5; q = 0.017) and a non-significant numerical increase in non-starchy vegetables (+55.0 g/day; 95% CI: −28.3 to 138.3), with a modest, non-significant rise in refined/starchy carbohydrates (+28.0 g/day; 95% CI: −20.3 to 76.4). Western Asia (WA) showed a statistically significant increase in seafood (+14.9 g/day; 95% CI: 11.2 to 18.5; q = 0.013), a non-significant decline in non-starchy vegetables (−8.7 g/day), and a non-significant rise in refined/starchy carbohydrates (+53.8 g/day). North Africa (NA) showed the largest absolute increase in refined/starchy carbohydrates (+155.4 g/day; 95% CI: −6.8 to 317.6), accompanied by a non-significant increase in seafood (+11.1 g/day) and a modest, non-significant decline in legumes (−3.2 g/day). Full numeric estimates and FDR-adjusted q-values are given in Supplementary Figure S1, Tables S1 and S2.

**Figure 4:**
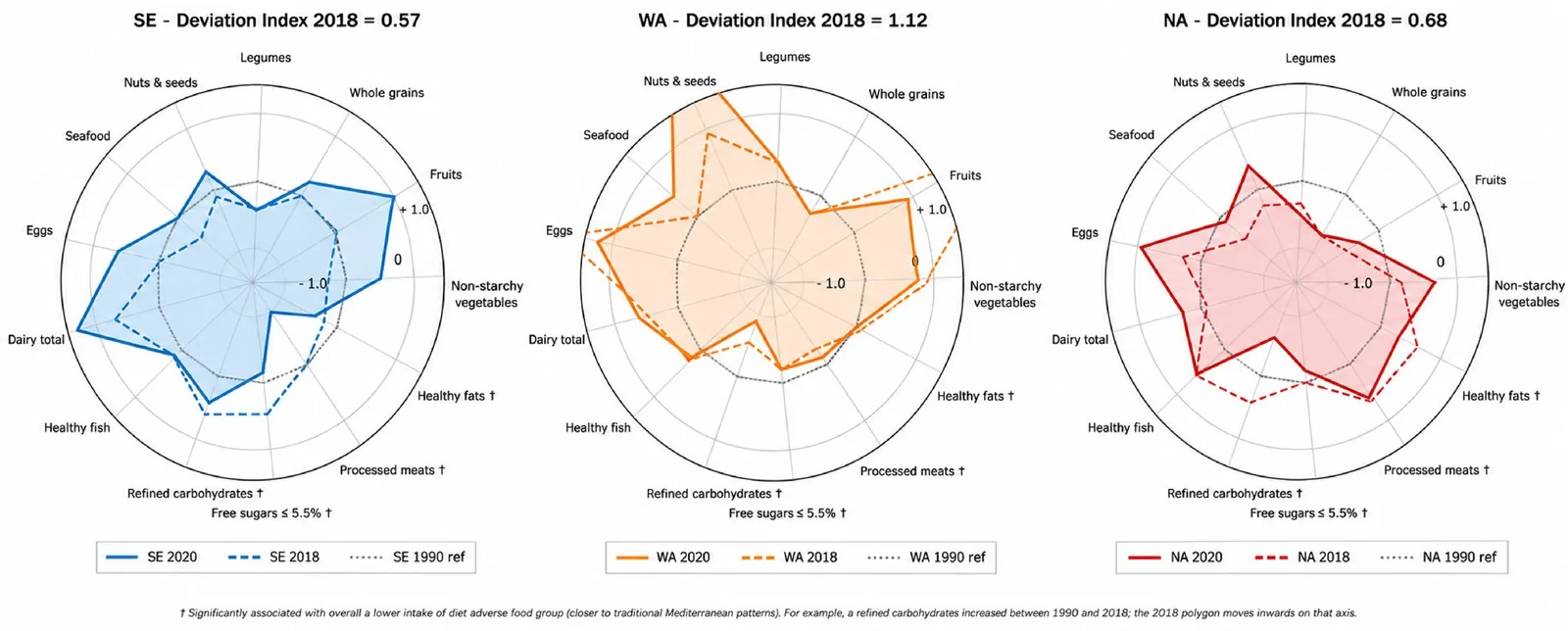
1990 → 2018 dietary trajectory by subregions (PSDD framework).

Considerable heterogeneity was observed across the three Mediterranean subregions in the magnitude and direction of dietary change between 1990 and 2018 (Tables S2 and S3; Figure 3 and Figure 4). Absolute intake values for 1990 and 2018, with percentage changes across all 13 food categories and three subregions, are provided in Supplementary Table S3.

Subregional dietary profiles relative to the SE 1990 reference pattern are visualised in Figure 3 and Figure 4 (PSDD radar). The qualitative ranking—SE showing the closest overall alignment with the reference profile, NA showing the largest single-axis deviation (refined/starchy carbohydrates), and WA showing the broadest multi-axis divergence—is supported by the per-food-group estimates reported above.

Seafood consumption increased significantly in Southern Europe (+13.71 g/day; 95% CI: 5.93–21.49; q = 0.017) and Western Asia (+14.86 g/day; 95% CI: 11.22–18.50; q = 0.013), while the increase in North Africa (+11.06 g/day; 95% CI: −1.65–23.78) did not reach statistical significance (q = 0.221).

Refined/starchy carbohydrates showed the largest absolute increase in North Africa (+155.37 g/day; 95% CI: −6.84 to 317.59), followed by Western Asia (+53.80 g/day; 95% CI: −51.38 to 158.97) and Southern Europe (+28.04 g/day; 95% CI: −20.28 to 76.36). None of these changes reached statistical significance after FDR correction; therefore, the North African estimate should be interpreted as a large but imprecise absolute change.

Non-starchy vegetables increased in Southern Europe (+55.01 g/day; 95% CI: −28.29–138.31) and North Africa (+34.57 g/day; 95% CI: −21.56–90.71), while declining in Western Asia (−8.65 g/day; 95% CI: −40.97–23.66). None reached statistical significance after FDR correction (q > 0.30 for all subregions).

Nuts and seeds increased significantly in Southern Europe (+2.73 g/day; 95% CI: 1.24–4.23; q = 0.017) and Western Asia (+12.28 g/day; 95% CI: 0.75–23.82), while the increase in North Africa (+4.34 g/day; 95% CI: −0.15–8.83) did not reach statistical significance. The apparent Western Asia increase did not, however, reach statistical significance after Benjamini–Hochberg FDR correction (q = 0.278); of the three subregions, only the Southern Europe increase remained significant after FDR correction (North Africa: q = 0.221).

Legumes declined modestly in North Africa (−3.17 g/day; 95% CI: −19.05–12.70) and Southern Europe (−0.25 g/day; 95% CI: −3.17–2.67), while showing a negligible increase in Western Asia (+0.30 g/day; 95% CI: −19.21–19.81). None of these changes were statistically significant.

Fruits increased in Southern Europe (+48.29 g/day; 95% CI: −46.00–142.58) and North Africa (+10.86 g/day; 95% CI: −10.58–32.30), while declining in Western Asia (−67.27 g/day; 95% CI: −260.28–125.75). None reached statistical significance.

Dairy total increased across all subregions: Southern Europe (+63.19 g/day; 95% CI: −4.11–130.50), North Africa (+42.25 g/day; 95% CI: −11.10–95.60), and Western Asia (+14.83 g/day; 95% CI: −10.01–39.68), without reaching statistical significance.

Eggs increased in North Africa (+6.47 g/day; 95% CI: 2.96–9.99) and Southern Europe (+6.23 g/day; 95% CI: −5.56–18.02), while declining in Western Asia (−5.06 g/day; 95% CI: −33.02–22.89).

Processed meats increased in Southern Europe (+16.88 g/day; 95% CI: 2.32–31.45) and North Africa (+1.18 g/day; 95% CI: −2.45–4.81), while declining in Western Asia (−3.53 g/day; 95% CI: −21.54–14.48).

Free sugars and sugar-sweetened beverages increased in Southern Europe (+77.15 g/day; 95% CI: −49.58–203.89) and North Africa (+25.01 g/day; 95% CI: −22.36–72.39), while showing a negligible change in Western Asia (−2.21 g/day; 95% CI: −85.79–81.37). None reached statistical significance.

Whole grains showed modest increases across all subregions: Southern Europe (+9.73 g/day; 95% CI: −35.32–54.78), North Africa (+1.73 g/day; 95% CI: −2.39–5.85), and Western Asia (+0.55 g/day; 95% CI: −4.98–6.09), without statistical significance.

MUFA (% kcal) showed minimal and non-significant changes across all subregions: North Africa (−0.26%; 95% CI: −2.99–2.47), Southern Europe (−0.06%; 95% CI: −1.29–1.16), and Western Asia (−0.29%; 95% CI: −3.96–3.38).

SFA + omega-6 PUFA (% kcal) increased modestly in all subregions: North Africa (+1.00%; 95% CI: −0.31–2.32), Southern Europe (+0.58%; 95% CI: −0.22–1.37), and Western Asia (+0.36%; 95% CI: −0.41–1.12), without statistical significance.

Subregional dietary profiles in 2018 were compared with the Southern Europe 1990 reference profile. Each food category was z-scaled against the 1990 cross-country distribution. Adverse categories (refined/starchy carbohydrates, free sugars and sugar-sweetened beverages, processed meats, and SFA + omega-6 PUFA) were sign-flipped so that outward displacement represents closer alignment with the traditional reference pattern. No RDI normalisation was applied.

Dietary trajectories from 1990 to 2018 for each Mediterranean subregion within the PSDD framework. Sensitivity analyses supported the primary findings: compositional (CLR) results were consistent with the absolute g/day changes, population-weighted estimates did not alter the qualitative conclusions, and energy-normalised (g/2000 kcal) analyses were qualitatively consistent with the primary results.

## 4. Discussion

Using harmonised dietary data from the GDD for 1990–2018, this study provides a novel population-level analysis of dietary change across Mediterranean coastline countries and comparator country groups. The findings indicate a divergent evolution of the Mediterranean dietary pattern: favourable elements, such as seafood, nuts, and some plant-based foods, increased in several settings, while unfavourable elements, such as refined/starchy carbohydrate intake, processed meats, free sugars, SSBs, and SFA + omega-6 PUFA, also increased in parts of the Mediterranean region. These findings suggest the coexistence of protective and adverse dietary shifts rather than simple preservation or uniform erosion of the traditional Mediterranean diet.

Critically, this transition was fragmented rather than uniform, with the balance between protective and adverse components differing markedly across Mediterranean subregions.

Compositional analyses supported this interpretation by showing that relative dietary shares changed in ways that were not captured by absolute g/day values alone. However, the CLR results should be interpreted as complementary sensitivity analyses rather than as independent evidence of dietary adequacy or healthfulness.

These findings are consistent with classical and emerging evidence describing the global nutrition transition, characterised by increased availability and consumption of ultra-processed foods, refined grains and added sugars [10,21]. Recent evidence also confirms declining adherence to the Mediterranean diet within parts of the Mediterranean region itself [22]. This decline is not only quantitative but also qualitative, reflecting partial replacement of traditional foods with refined/starchy carbohydrates and ultra-processed products.

A key contribution of the present study is the identification of substantial regional heterogeneity. Southern Europe appeared comparatively resilient, with statistically significant increases in seafood and increases in vegetables, alongside smaller rises in refined/starchy carbohydrates. This aligns with earlier evidence of relatively higher adherence in countries such as Italy, Spain and Greece [13,14,23]. However, recent data suggest that even these regions may be experiencing gradual declines in diet quality or Mediterranean diet adherence, particularly among younger populations [24,25,26]. Systematic reviews corroborate this broader trend across Mediterranean countries, noting that generational dietary shifts represent a growing public health concern even in relatively resilient settings such as Southern Europe [22,24].

North Africa showed the largest absolute increase in refined/starchy carbohydrate intake (+155.4 g/day), although this estimate was imprecise and not statistically significant after FDR correction. This finding is consistent with the possibility that lower- and middle-income Mediterranean regions may be vulnerable to dietary westernisation under economic constraints, food-price volatility and reliance on low-cost, energy-dense staples [27]. Western Asia, despite a smaller absolute increase in refined/starchy carbohydrates, registered the greatest overall divergence from the Southern Europe 1990 reference profile. These patterns indicate that the Mediterranean dietary transition is shaped not only by absolute food-group changes, but also by the changing balance between protective and adverse food groups under urbanisation, globalisation, and changing food environments.

Changes in fatty acid composition, including MUFA and SFA + omega-6 PUFA, appeared modest and statistically non-significant across Mediterranean subregions. This suggests that the recent dietary shifts in the present dataset are driven primarily by changes in food-group composition rather than by large changes in fatty-acid composition alone. Given the central role of olive oil and MUFA-rich foods in the traditional Mediterranean diet, the relative stability of MUFA may indicate partial preservation of dietary identity despite broader structural changes.

The observed dietary shifts have important public health implications. Higher adherence to the Mediterranean diet is associated with reduced all-cause mortality and lower risk of cardiovascular and metabolic diseases [28]. Conversely, westernised dietary patterns characterised by ultra-processed foods, refined/starchy carbohydrates and added sugars are associated with obesity, type 2 diabetes and cardiovascular disease risk [10,21,25]. These opposing trends underscore a public health paradox: while the Mediterranean diet is globally promoted as a model of healthy eating, adherence within parts of the region itself appears to be declining.

From a policy perspective, these findings underscore the need for targeted, region-specific interventions to address the structural drivers of dietary change. Evidence indicates that adherence to the Mediterranean diet is shaped by multi-level determinants, including socio-economic conditions, food environments and policy frameworks [27]. Effective strategies should therefore extend beyond individual behaviour change to include structural interventions, such as improving access to affordable healthy foods, regulating ultra-processed food environments, and supporting traditional food systems. The marked heterogeneity across subregions further emphasises that a one-size-fits-all approach is unlikely to be effective. Policy responses could draw on existing frameworks, including the WHO European Food and Nutrition Action Plan 2015–2020 and national Mediterranean diet promotion initiatives in countries such as Italy, Spain, and Greece, adapting these to the specific socio-economic and food-environment contexts of North Africa and Western Asia.

This study has several strengths, including the use of harmonised global dietary data across a large number of countries and the application of both absolute and compositional analytical approaches. However, limitations should be acknowledged. These include reliance on country-level estimates—which cannot support individual-level inference and are subject to ecological fallacy—potential heterogeneity in dietary assessment methods, inability to capture within-country inequalities (by socio-economic status, urban/rural residence, or age group) or individual-level dietary behaviours, absence of harmonised unprocessed meat estimates, the heterogeneous composition of the refined/starchy carbohydrate category, and the exploratory, descriptive nature of the PSDD radar framework, which was used here for visualisation only and is not a validated Mediterranean-diet adherence score. In addition, the fatty acid composition data does not identify the food source. Different sources of MUFA have different health effects. Although MED regions may consume predominantly olive oil (assumed), meat is also a rich source of MUFA. The SATF and N6 sources will also vary in source. Furthermore, country-level data preclude acknowledgement of dietary combinations at the individual level, which may have synergistic health benefits or adverse effects. Additionally, we do not have any information on processing methods—e.g., for nuts and seeds, we do not know whether salt was added; for meat, we do not know whether fat was removed, among other processes. Moreover, cooking practices and food preparation methods vary across countries for cultural reasons and may have changed between 1990 and 2018. Finally, the GDD food categories do not map directly onto the NOVA ultra-processed food classification; future analyses incorporating NOVA-aligned data would complement the present findings by capturing food processing level as an additional dietary dimension.

## 5. Conclusions

In conclusion, Mediterranean countries are undergoing a fragmented nutrition transition characterised by partial preservation of traditional dietary elements alongside increasing westernisation. Southern Europe showed the closest alignment with the traditional reference profile, Western Asia showed the greatest overall divergence, and North Africa showed the largest absolute increase in refined/starchy carbohydrate intake. Protective dietary elements increased concurrently with adverse dietary shifts, suggesting coexistence rather than straightforward preservation of the traditional Mediterranean pattern. These findings support targeted, region-specific public health strategies to preserve beneficial Mediterranean dietary components while addressing emerging diet-related risks.

## Figures and Tables

**Table 1:** The Global Food Category Definitions.

Category	Components Included	Database Source Column(s)	Units
Non-starchy vegetables	Non-starchy vegetables; salads and leafy greens; fresh herbs	Non-starchy vegetables	g/day
Fruits	Whole fruits (fresh, frozen, dried); all fruit varieties; excludes fruit juices	Fruits	g/day
Whole grains	Brown rice; whole wheat bread/pasta; oats, quinoa; other intact grains	Whole grains	g/day
Legumes	Beans (black, kidney, navy, etc.); lentils and chickpeas; peas and other pulses	Beans and legumes	g/day
Nuts & seeds	Tree nuts (almonds, walnuts, etc.); seeds (sunflower, pumpkin, etc.); nut and seed butters	Nuts and seeds	g/day
Seafood	Fish (all types); shellfish and mollusks; other marine foods	Total seafoods	g/day
Eggs	Chicken eggs; other bird eggs; all preparations	Eggs	g/day
Dairy total	Cheese (all types); yoghurt (including fermented milk); milk (fluid milk, all fat levels)	Cheese + Yoghurt + Total Milk	g/day
Processed meats	Bacon and sausages; deli/lunch meats; cured and smoked meats; hot dogs and frankfurters	Total processed meats	g/day
Refined/starchy carbohydrates	Refined grains (white bread, white rice, white pasta); potatoes (all preparations); other starchy vegetables (corn, cassava, etc.)	Refined grains) + Potatoes+ Other starchy vegetables	g/day
Free sugars & beverages	Sugar-sweetened beverages (sodas, sports drinks, energy drinks); fruit juices (100% fruit juice, all varieties)	Sugar-sweetened beverages + Fruit juices	g/day
MUFA	Monounsaturated fatty acids; primary sources: olive oil, avocados; secondary: nuts, certain plant oils	Monounsaturated fatty acids	% total kcal/day
SFA + omega-6 PUFA	Saturated fat (animal fats, tropical oils); omega-6 fatty acids (industrial seed oils, processed foods); sources: margarine, fried foods, packaged snacks	Saturated fat (% of total kcal) + Total omega-6 fat	% total kcal

Note on meat categories. The GDD provides total processed meats but does not provide separate country-year estimates for unprocessed red or white meats with the same level of harmonisation. Unprocessed meat is therefore not analysed as a stand-alone food group in this study and should not be inferred directly from fatty-acid composition. Its contribution is only partially reflected in total energy intake and fatty-acid patterns; this limitation is acknowledged in the Discussion.

## Data Availability

The raw data used in this study are openly available in the Global Dietary Database at https://www.globaldietarydatabase.org (accessed on 1 July 2025). Derived datasets and analysis code are available from the corresponding author upon reasonable request.

## Supplementary Materials

The following supporting information can be downloaded at https://www.mdpi.com/article/10.3390/nu18182943/s1. Figure S1, Mediterranean subregion within-country change in food group intake (g/day), 1990–2018. Point estimates with 95% CI. Table S1: Within-group dietary change with unadjusted p-values and Cohen’s d effect sizes, by Mediterranean subregion; Table S2: Mediterranean subregion FDR-adjusted q-values (g/day); Table S3: Absolute food group intake in 1990 and 2018, with percentage change, across three Mediterranean subregions; Table S4: Country classification used in the analysis.
