# Supplementary material for: Mediterranean Diet and Global Dietary Shifts, 1990–2018: Population-Level Analysis Using Global Dietary Database
Source: Nutrients. 2026 Sep 8;18(18):2943. doi: 10.3390/nu18182943 (PMC13610221; doi:10.3390/nu18182943)
Supplement: Supplementary file 1 [file nutrients-18-02943-s001.zip › nutrients-4377172-supplementary.pdf]

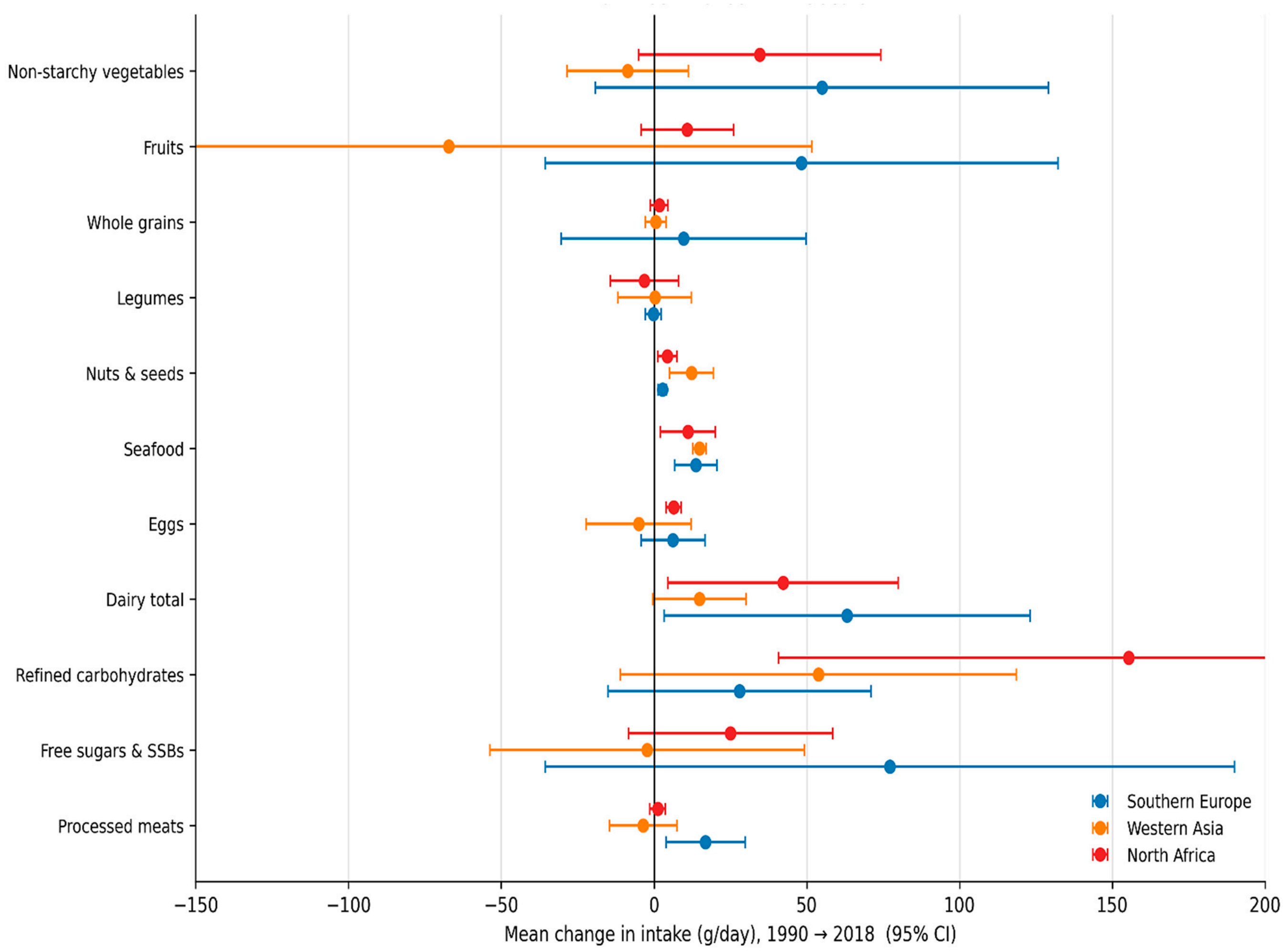

Supplementary Figure S1 Mediterranean subregion within-country change in food group intake (g/day), 1990–2018. Point estimates with 95% CI.

Supplementary Table S1. Within-group dietary change with unadjusted p-values and Cohen's d effect sizes, by Mediterranean subregion.

Food Category	Subregion	n	Mean Δ	SE	95% CI Lower	95% CI Upper	p-value	Cohen's d
Non-starchy vegetables (g/day)	Southern Europe	12	55.01	37.85	-28.29	138.31	0.1740	0.420
Non-starchy vegetables (g/day)	Western Asia	4	-8.65	10.15	-40.97	23.66	0.4567	-0.426
Non-starchy vegetables (g/day)	North Africa	5	34.57	20.22	-21.56	90.71	0.1624	0.765
Fruits (g/day)	Southern Europe	12	48.29	42.84	-46.00	142.58	0.2836	0.325
Fruits (g/day)	Western Asia	4	-67.27	60.65	-260.28	125.75	0.3483	-0.555
Fruits (g/day)	North Africa	5	10.86	7.72	-10.58	32.30	0.2323	0.629
Whole grains (g/day)	Southern Europe	12	9.73	20.47	-35.32	54.78	0.6437	0.137
Whole grains (g/day)	Western Asia	4	0.55	1.74	-4.98	6.09	0.7713	0.159
Whole grains (g/day)	North Africa	5	1.73	1.48	-2.39	5.85	0.3086	0.521
Legumes (g/day)	Southern Europe	12	-0.25	1.33	-3.17	2.67	0.8533	-0.055
Legumes (g/day)	Western Asia	4	0.30	6.13	-19.21	19.81	0.9638	0.025
Legumes (g/day)	North Africa	5	-3.17	5.72	-19.05	12.70	0.6084	-0.248
Nuts & seeds (g/day)	Southern Europe	12	2.73	0.68	1.24	4.23	0.0020	1.161
Nuts & seeds (g/day)	Western Asia	4	12.28	3.62	0.75	23.82	0.0428	1.694
Nuts & seeds (g/day)	North Africa	5	4.34	1.62	-0.15	8.83	0.0550	1.201
Seafood (g/day)	Southern Europe	12	13.71	3.54	5.93	21.49	0.0026	1.119
Seafood (g/day)	Western Asia	4	14.86	1.14	11.22	18.50	0.0010	6.494

Seafood (g/day)	North Africa	5	11.06	4.58	-1.65	23.78	0.0731	1.080
Eggs (g/day)	Southern Europe	12	6.23	5.36	-5.56	18.02	0.2694	0.336
Eggs (g/day)	Western Asia	4	-5.06	8.78	-33.02	22.89	0.6048	-0.288
Eggs (g/day)	North Africa	5	6.47	1.27	2.96	9.99	0.0069	2.288
Dairy (milk + yoghurt + cheese, g/day)	Southern Europe	12	63.19	30.58	-4.11	130.50	0.0632	0.597
Dairy (milk + yoghurt + cheese, g/day)	Western Asia	4	14.83	7.81	-10.01	39.68	0.1536	0.950
Dairy (milk + yoghurt + cheese, g/day)	North Africa	5	42.25	19.22	-11.10	95.60	0.0928	0.983
Processed meats (g/day)	Southern Europe	12	16.88	6.62	2.32	31.45	0.0270	0.736
Processed meats (g/day)	Western Asia	4	-3.53	5.66	-21.54	14.48	0.5769	-0.312
Processed meats (g/day)	North Africa	5	1.18	1.31	-2.45	4.81	0.4180	0.403
Refined carbohydrates (g/day)	Southern Europe	12	28.04	21.95	-20.28	76.36	0.2278	0.369
Refined carbohydrates (g/day)	Western Asia	4	53.80	33.05	-51.38	158.97	0.2020	0.814
Refined carbohydrates (g/day)	North Africa	5	155.37	58.43	-6.84	317.59	0.0564	1.189
Free sugars & sugary drinks (g/day)	Southern Europe	12	77.15	57.58	-49.58	203.89	0.2073	0.387
Free sugars & sugary drinks (g/day)	Western Asia	4	-2.21	26.26	-85.79	81.37	0.9381	-0.042
Free sugars & sugary drinks (g/day)	North Africa	5	25.01	17.06	-22.36	72.39	0.2165	0.656
Healthy fats (MUFA % energy)	Southern Europe	12	-0.06	0.56	-1.29	1.16	0.9137	-0.032
Healthy fats (MUFA % energy)	Western Asia	4	-0.29	1.15	-3.96	3.38	0.8174	-0.126

Healthy fats (MUFA % energy)	North Africa	5	-0.26	0.98	-2.99	2.47	0.8040	-0.119
Unhealthy fats (Sat + Omega-6 % energy)	Southern Europe	12	0.58	0.36	-0.22	1.37	0.1404	0.459
Unhealthy fats (Sat + Omega-6 % energy)	Western Asia	4	0.36	0.24	-0.41	1.12	0.2339	0.743
Unhealthy fats (Sat + Omega-6 % energy)	North Africa	5	1.00	0.47	-0.31	2.32	0.1019	0.946

Supplementary Table S2. Mediterranean subregion FDR-adjusted q-values (g/day).

Outcome	North Africa	Southern Europe	Western Asia
Dairy (milk+yoghurt+cheese)	0.2207	0.2052	0.6081
Eggs	0.0898	0.3687	0.8736
Free sugars & SSBs	0.3356	0.3687	0.9638
Fruits	0.3356	0.3687	0.7546
MUFA (% kcal)	0.8040	0.9137	0.9638
Legumes	0.6591	0.9137	0.9638
Non-starchy vegetables	0.3016	0.3687	0.8481
Nuts & seeds	0.2207	0.0167	0.2783
Processed meats	0.4940	0.1168	0.8736
Refined/starchy carbohydrates	0.2207	0.3687	0.6081
Seafood	0.2207	0.0167	0.0128
SFA + omega-6 PUFA (% kcal)	0.2207	0.3650	0.6081
Whole grains	0.4012	0.7608	0.9638

Supplementary Table S3. Absolute food group intake in 1990 and 2018, with percentage change, across three Mediterranean subregions.

Food Category	Unit	SE 1990	SE 2018	SE % Δ	WA 1990	WA 2018	WA % Δ	NA 1990	NA 2018	NA % Δ
Non-starchy vegetables	g/day	109.3 ± 26.2	164.3 ± 123.9	+50.3%	200.3 ± 110.9	191.6 ± 123.3	-4.3%	146.6 ± 27.9	181.2 ± 38.1	+23.6%
Fruits	g/day	99.7 ± 66.5	148.0 ± 117.5	+48.5%	212.9 ± 175.0	145.6 ± 54.8	-31.6%	72.8 ± 18.4	83.7 ± 22.7	+14.9%
Whole grains	g/day	39.8 ± 46.3	49.6 ± 52.2	+24.4%	26.4 ± 23.1	27.0 ± 21.6	+2.1%	11.9 ± 6.3	13.6 ± 7.5	+14.6%
Legumes	g/day	18.8 ± 9.2	18.6 ± 11.0	-1.3%	35.2 ± 16.4	35.5 ± 21.5	+0.9%	21.2 ± 15.8	18.0 ± 6.5	-15.0%
Nuts & seeds	g/day	3.6 ± 2.9	6.3 ± 2.3	+76.2%	10.6 ± 8.4	22.9 ± 12.7	+115.9%	2.5 ± 1.9	6.8 ± 4.4	+173.2%
Seafood	g/day	18.3 ± 15.2	32.0 ± 17.6	+75.1%	32.0 ± 46.9	46.8 ± 48.2	+46.4%	16.7 ± 8.1	27.7 ± 9.0	+66.4%
Eggs	g/day	16.6 ± 4.7	22.9 ± 18.7	+37.5%	34.2 ± 20.2	29.1 ± 16.3	-14.8%	19.3 ± 3.7	25.8 ± 4.2	+33.5%
Dairy (milk + yoghurt + cheese)	g/day	218.6 ± 49.5	281.8 ± 108.7	+28.9%	194.3 ± 85.7	209.1 ± 70.1	+7.6%	128.9 ± 44.0	171.1 ± 58.4	+32.8%
Processed meats	g/day	17.6 ± 10.0	34.4 ± 28.6	+96.2%	22.1 ± 32.1	18.6 ± 20.8	-16.0%	4.8 ± 3.0	5.9 ± 4.9	+24.8%
Refined/starchy carbohydrates	g/day	235.5 ± 94.4	263.5 ± 113.7	+11.9%	405.4 ± 76.2	459.2 ± 34.3	+13.3%	264.6 ± 64.9	420.0 ± 126.1	+58.7%
Free sugars & sugary drinks	g/day	90.3 ± 42.3	167.5 ± 195.0	+85.4%	172.5 ± 78.6	170.3 ± 44.2	-1.3%	149.2 ± 37.0	174.3 ± 42.1	+16.8%
Healthy fats (MUFA % energy)	% kcal	12.6 ± 3.9	12.5 ± 2.3	-0.5%	13.2 ± 6.8	12.9 ± 6.6	-2.2%	14.8 ± 3.3	14.5 ± 2.4	-1.8%
Unhealthy fats (Sat + ω-6 % energy)	% kcal	14.6 ± 2.1	15.1 ± 2.0	+4.0%	13.5 ± 2.8	13.9 ± 2.4	+2.6%	11.7 ± 2.5	12.7 ± 2.5	+8.5%

Note: Values are mean ± SD of country-level intakes within each subregion. SE = Southern Europe (n = 12); WA = Western Asia (n = 4); NA = North Africa (n = 5). % Δ = percentage change from 1990 to 2018. g/day categories are absolute intake; fat categories are expressed as % of total energy (kcal).

Supplementary Table S4. Country classification used in the analysis.

Group	n	Countries
Mediterranean coastline countries (MED)	21	Albania; Algeria; Bosnia and Herzegovina; Croatia; Cyprus; Egypt; France; Greece; Israel; Italy; Lebanon; Libya; Malta; Montenegro; Morocco; Palestine; Slovenia; Spain; Syria; Tunisia; Türkiye
High-income non-Mediterranean countries (HIC non-MED)	18	Australia; Austria; Belgium; Canada; Denmark; Finland; Germany; Iceland; Ireland; Japan; Netherlands; New Zealand; Norway; Republic of Korea; Sweden; Switzerland; United Kingdom; United States
Middle- and low-income non-Mediterranean countries (MLIC non-MED)	146	Afghanistan; Angola; Argentina; Armenia; Azerbaijan; Bangladesh; Belarus; Belize; Benin; Bhutan; Bolivia; Botswana; Brazil; Bulgaria; Burkina Faso; Burundi; Cambodia; Cameroon; Central African Republic; Chad; Chile; China; Colombia; Comoros; Congo; Costa Rica; Côte d'Ivoire; Cuba; Democratic Republic of the Congo; Djibouti; Dominican Republic; Ecuador; El Salvador; Equatorial Guinea; Eritrea; Eswatini; Ethiopia; Fiji; Gabon; Gambia; Georgia; Ghana; Guatemala; Guinea; Guinea-Bissau; Guyana; Haiti; Honduras; India; Indonesia; Iran; Iraq; Jamaica; Jordan; Kazakhstan; Kenya; Kosovo; Kyrgyzstan; Lao People's Democratic Republic; Lesotho; Liberia; Madagascar; Malawi; Malaysia; Maldives; Mali; Mauritania; Mauritius; Mexico; Moldova; Mongolia; Mozambique; Myanmar; Namibia; Nepal; Nicaragua; Niger; Nigeria; North Macedonia; Pakistan; Panama; Papua New Guinea; Paraguay; Peru; Philippines; Romania; Russian Federation; Rwanda; São Tomé and Príncipe; Senegal; Serbia; Sierra Leone; Somalia; South Africa; Sri Lanka; Sudan; Suriname; Tajikistan; Tanzania; Thailand; Timor-Leste; Togo; Turkmenistan; Uganda; Ukraine; Uruguay; Uzbekistan; Venezuela; Viet Nam; Yemen; Zambia; Zimbabwe
